# Melatonin suppresses TLR9-triggered proinflammatory cytokine production in macrophages by inhibiting ERK1/2 and AKT activation

**DOI:** 10.1038/s41598-018-34011-8

**Published:** 2018-10-22

**Authors:** Xiongfei Xu, Guoquan Wang, Lingling Ai, Jianhui Shi, Jing Zhang, Yu-Xia Chen

**Affiliations:** 1 Department of Pathophysiology, Second Military Medical University, Shanghai, 200433 China; 2 Department of Hepatobiliary Pancreatic Surgery, Changhai Hospital, Second Military Medical University, Shanghai, 200433 China; 3 Department of Neurology, Junkang Hospital, Shanghai, 200125 China; 4 Department of Otolaryngology, No. 455 Hospital of PLA, Shanghai, 200052 China; 5 Department of Pathology, Changhai Hospital, Second Military Medical University, Shanghai, 200433 China

## Abstract

Toll-like receptor (TLR) signaling plays major roles in innate immune response in macrophages. Melatonin regulates TLR3- and TLR4-mediated innate immune responses in macrophages. However, it remains unknown whether melatonin regulates TLR9-mediated innate immune responses in macrophages. Here we demonstrated that melatonin suppressed TLR9 ligand-induced proinflammatory cytokines mRNA and protein production in peritoneal macrophages without interrupting the viability of peritoneal macrophages. Using a melatonin membrane receptors MT1/MT2 antagonist luzindole, we found that MT1 and MT2 were dispensable for melatonin’s inhibitory effects on TLR9-mediated proinflammatory cytokines production, even though melatonin upregulated mRNA expression of MT1 and MT2 in macrophages. Furthermore, melatonin did not affect mRNA expressions of TLR9 and MyD88 but attenuated TLR9 ligand-induced ERK1/2 and AKT phosphorylation without affecting p38 and NF-κB p65 phosphorylation. Also, melatonin inhibited TLR9-mediated proinflammatory cytokines production *in vivo*. Taken together, our results demonstrate that melatonin suppresses TLR9-triggered proinflammatory cytokines production in macrophages via melatonin membrane receptor-independent manners and probably through inhibiting ERK1/2 and AKT activation, which further elucidates the roles of melatonin in regulating TLR-mediated innate immune responses in macrophages.

## Introduction

Toll-like receptor (TLR), an important pattern-recognition receptor (PRR), is critical for host defence against invading pathogens^1,2^. TLRs initiate innate immune responses through activating innate immune cells such as macrophages and dendritic cells (DCs)^1,2^. TLR family members recognize different components of pathogens, which are referred to as pathogen-associated molecular patterns (PAMPs), and each TLR has a distinct function in terms of PAMP recognition and immune responses^1,3^. For instance, TLR4, TLR3 and TLR9 recognize lipopolysaccharide (LPS) from gram-negative bacteria, double-stranded RNA from virus, and CpG DNA from bacteria and viruses, respectively^1,3^. Ligation of TLRs by PAMPs induces the activation of intracellular signaling molecules such as MyD88 and TRIF, which in turn lead to the production of nuclear factor-κB (NF-κB) and mitogen-activated protein kinases (MAPKs)-dependent pro-inflammatory cytokines, or the production of interferon regulatory factor (IRF)-dependent type I interferons (IFNs)^1–3^. With regard to TLR9, it undergoes proteolytic cleavage in endosomes after ligation by CpG DNA; the cleaved TLR9 then recruits MyD88 to activate NF-κB and MAPKs and to induce production of pro-inflammatory cytokines, or induce production of type I IFN by activating IRF7^1,3^. Although TLRs are essential for host protective immunity against pathogens, inappropriate TLRs activation can lead to prolonged inflammation and even autoimmune and inflammatory diseases^1,2^. Hence, signaling molecules or substances that fine-tune TLR pathways have been receiving increased attention in recent years. Many molecules have been found to negatively regulate TLR signaling by different mechanisms. These molecules include cell membrane or intracellular molecules, and even intranuclear epigenetic modifiers^2,4–7^. In a previous study, Stk38 protein kinase was found to preferentially inhibit TLR9-activated inflammatory responses by promoting MEKK2 ubiquitination in macrophages^4^. Furthermore, various substances have been found to regulate TLR signaling, such as micheliolide, vasoactive intestinal peptide, and melatonin^8–12^.

Melatonin is mainly produced by the pineal gland, but extrapineal tissues, such as gastrointestinal tract, retina, spleen, liver and kidney, and endothelial cells or immune cells can also produce melatonin^13,14^. Melatonin has a wide range of functions, which are divided into chronobiotic and nonchronobiotic ones^13^. The chronobiotic effects regulate the circadian rhythms and seasonal changes, and are mediated by melatonin in the circulation due to nocturnal pineal synsthesis, whereas melatonin produced by other cells, such as gastrointestinal and immune cells, is independent of the light/dark cycle and exerts nonchronobiotic effects, including antioxidant, oncostatic, antiaging, and immunomodulatory effects^13^.

The effects of melatonin in the body are mediated mainly by two pathways: melatonin receptor-dependent pathway and melatonin receptor-independent pathway^14,15^. Melatonin receptors include membrane receptors MT1/MT2, and nuclear receptors RORα/RZR^14,15^. Increasing evidences demonstrate that melatonin plays a fundamental role in regulating immune response, as well as infection, sepsis, inflammation, and autoimmunity^13,15–18^. Recently, more and more studies show that melatonin modulates the functions of innate immune cells such as macrophages, monocytes, natural killer cells and neutrophils^13,17^. In particular, melatonin regulates TLR3 and TLR4 signalings in macrophages^8–10,13,17^. However, it remains to be elucidated whether melatonin regulates TLR9-mediated innate immune responses in macrophages.

In this study, we observed that melatonin suppressed TLR9 ligand-induced proinflammatory cytokines production in primary peritoneal macrophages. Moreover, its suppressive effects on TLR9 signaling did not depend on melatonin membrane receptors MT1 and MT2, but probably through inhibiting ERK1/2 and AKT activation without affecting mRNA expressions of TLR9 and MyD88. Furthermore, melatonin also decreased TLR9-mediated TNF-α and IL-6 production in mouse serum. Overall, the results of our study clarify the inhibitory effects of melatonin on TLR9-mediated innate immune responses in macrophages, probably through suppressing ERK1/2 and AKT activation.

## Results

### Melatonin suppresses TLR9 ligand-induced proinflammatory cytokines production in macrophages

**Figure 1:**
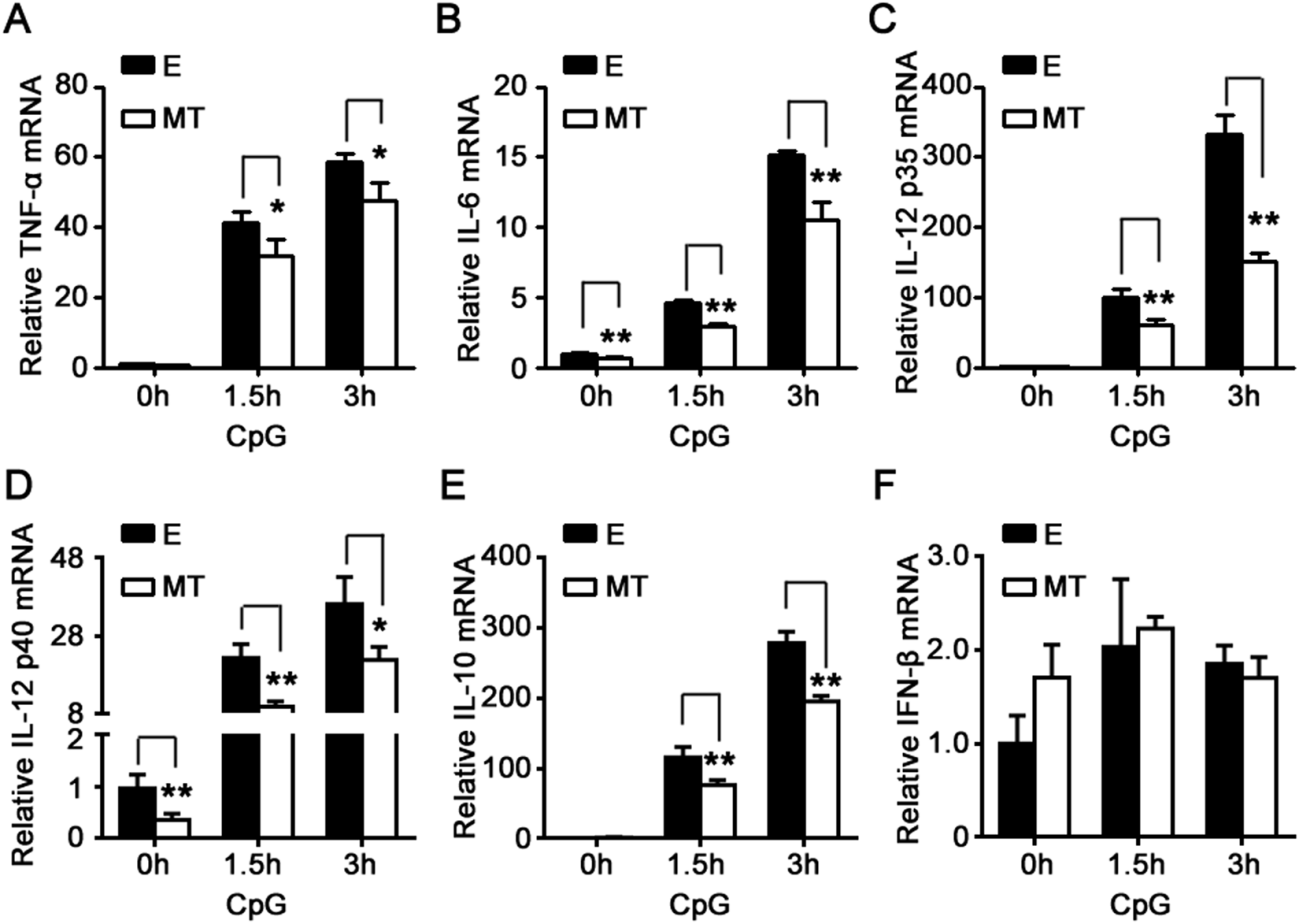
The effects of melatonin on mRNA expressions of cytokines mediated by TLR9 in peritoneal macrophages. Peritoneal macrophages were pre-treated with 1 mM MT or solvent control (ethanol, E) for 1 hour and then stimulated with 2 μg/ml CpG-ODN for 0, 1.5 or 3 hours. TNF-α (**A**), IL-6 (**B**), IL-12 p35 (**C**) IL-12 p40 (**D**), IL-10 (**E**) and IFN-β (**F**) mRNA expressions were detected by real-time PCR. Data are shown as mean ± s.d. (n = 3). ^*^P < 0.05, ^**^P < 0.01 (Student’s t-test). Similar results were obtained in three independent experiments.

To investigate whether melatonin could regulate TLR9-mediated inflammatory responses in macrophages, primary peritoneal macrophages were pre-treated with melatonin and then stimulated with TLR9 ligand CpG-ODN. First, we investigated the effects of melatonin on mRNA expressions of inflammatory cytokines in macrophages induced by CpG-ODN. As shown in Fig. 1A–F, CpG-ODN induced TNF-α, IL-6, IL-12 p35, IL-12 p40 and IL-10 mRNA expressions promptly, while IFN-β mRNA expression were not increased significantly after CpG-ODN stimulation. Interestingly, 1 mM melatonin significantly attenuated CpG-ODN-induced upregulation of TNF-α, IL-6, IL-12 p35, IL-12 p40 and IL-10 mRNA expressions but did not affect IFN-β mRNA expression (Fig. 1A–F), demonstrating that melatonin could suppress mRNA expressions of CpG-ODN-induced inflammatory cytokines.

**Figure 2:**
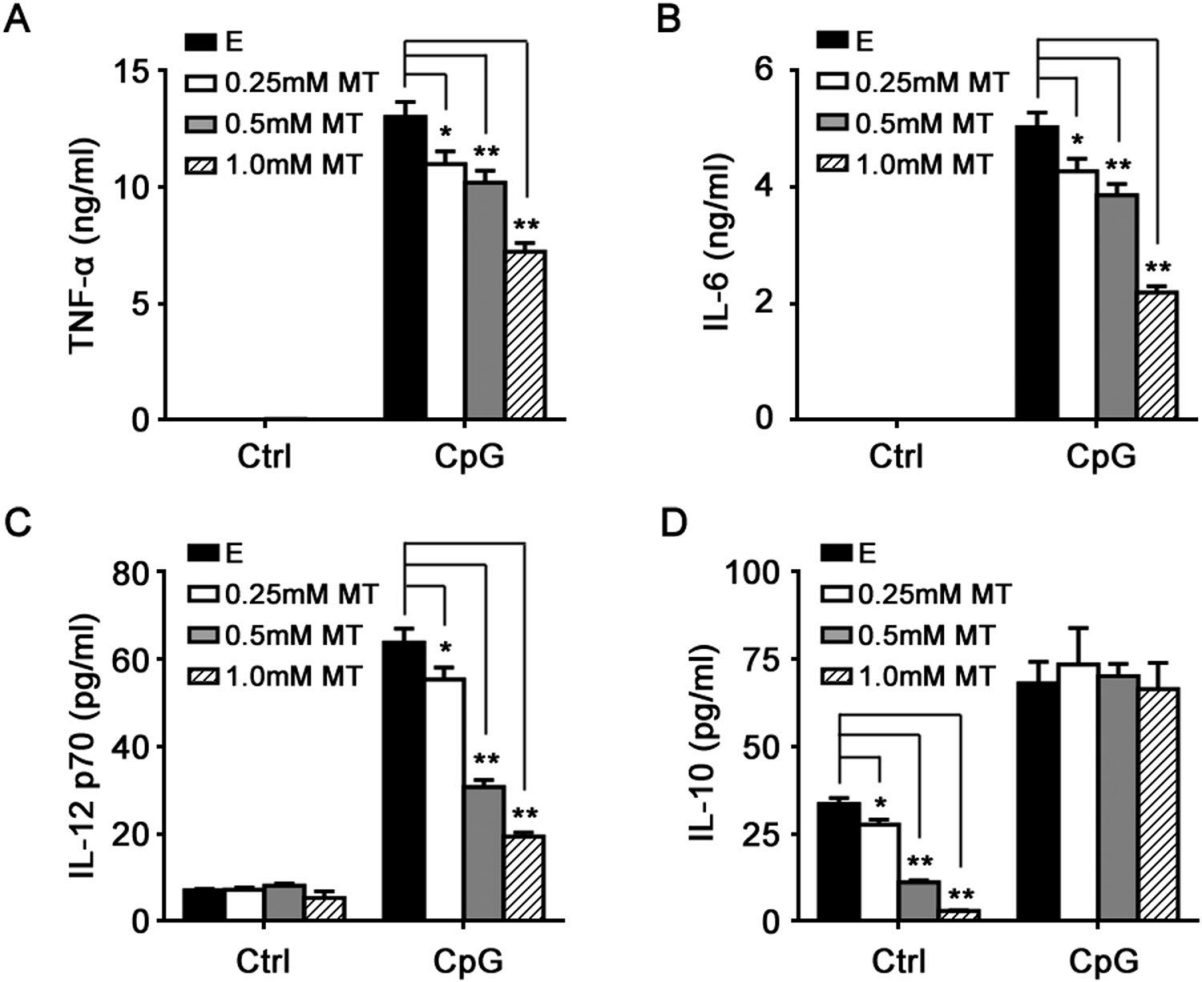
Melatonin suppresses TLR9-mediated inflammatory cytokines production in peritoneal macrophages. Peritoneal macrophages were pre-treated with various concentrations of MT or E for 1 hour and then stimulated with 2 μg/ml CpG-ODN for 24 hours. TNF-α (**A**), IL-6 (**B**), IL-12 p70 (**C**) and IL-10 (**D**) in the supernatants were assayed by ELISA. Data are shown as mean ± s.d. (n = 3). ^*^P < 0.05, ^**^P < 0.01 (ANOVA). Similar results were obtained in three independent experiments.

Then we assessed the effects of melatonin on protein expression of inflammatory cytokines in macrophage induced by CpG-ODN. As shown in Fig. 2A–D, 2 μg/ml CpG-ODN significantly induced the production of TNF-α, IL-6, IL-12 p70 and IL-10 in macrophages, whereas, as expected, it induced no production of INF-β (Data not shown). Melatonin effectively suppressed the production of TNF-α, IL-6 and IL-12 p70 induced by CpG-ODN in a dose-dependent manner (Fig. 2A–C), whereas it exerted no notable suppression on anti-inflammatory IL-10 production (Fig. 2D). Of note, melatonin alone could inhibit the constitutive IL-10 production in macrophages (Fig. 2D). The results above indicate that melatonin could markedly suppress TLR9-mediated inflammatory responses in macrophages.

Notably, melatonin inhibited IL-10 mRNA expression but not IL-10 protein expression in CpG-ODN-stimulated macrophages (Figs 1E and 2D), indicating the possible posttranscriptional regulation by melatonin. The result also showed that melatonin suppressed TLR9-mediated proinflammatory cytokines production in macrophages not through upregulation of anti-inflammatory IL-10.

**Figure 3:**
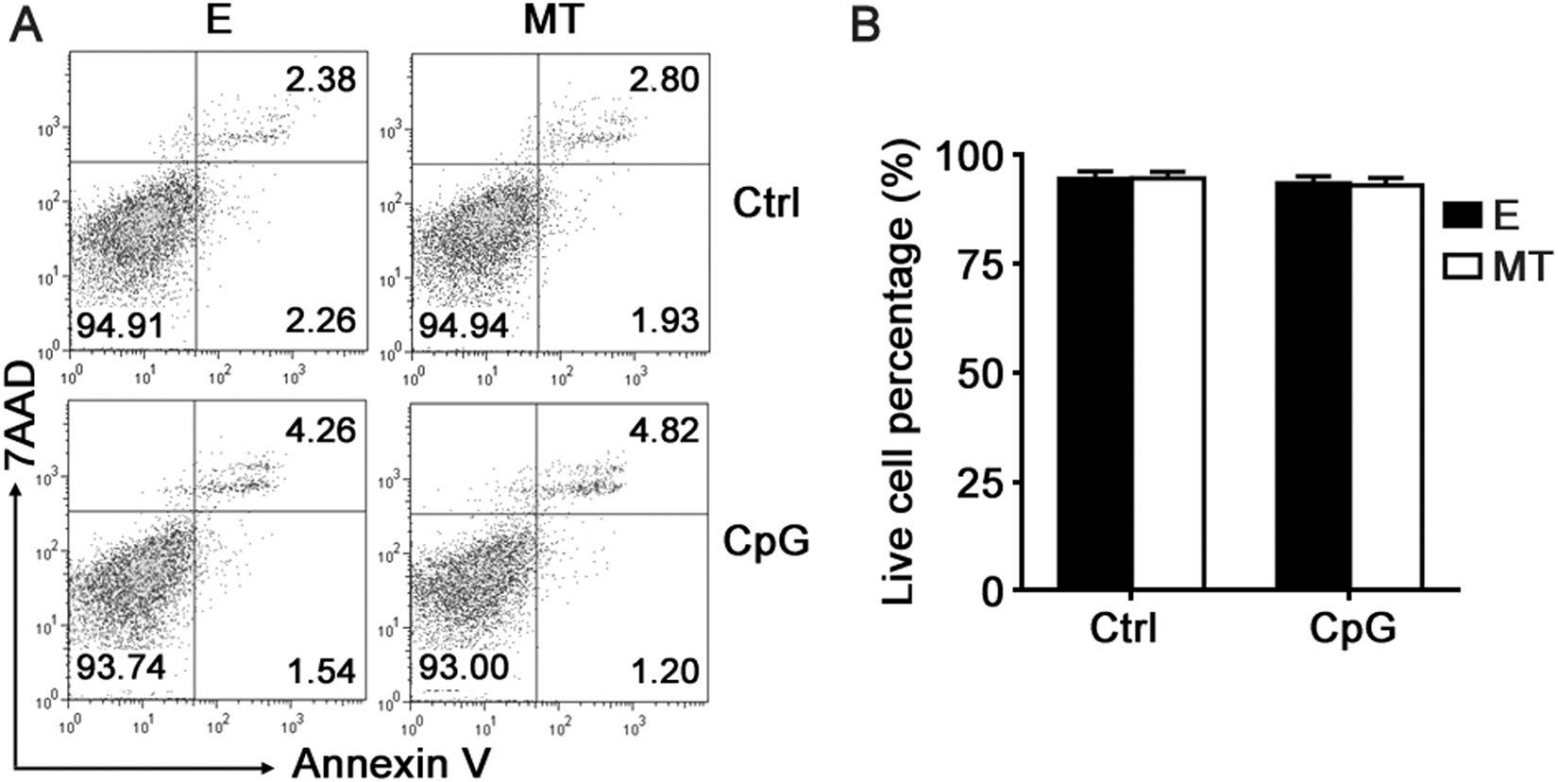
Melatonin does not influence the viability of peritoneal macrophages. (**A**) Peritoneal macrophages were pre-treated with 1 mM MT or E for 1 hour and then stimulated with 2 μg/ml CpG-ODN for 24 hours. Cells were stained with annexin V/7AAD and the apoptosis was assayed by flow cytometry. Numbers in plots indicate the percentage of annexin V^−^ 7AAD^−^ cells (live cells), annexin V^+^ 7AAD^−^ cells (early apoptotic cells) and annexin V^+^ 7AAD^+^ cells (late apoptotic cells) in macrophages. (**B**) The percentage of live cells in A were analyzed statistically and summarized. Similar results were obtained in three independent experiments.

To exclude the possibility that melatonin may affect cell viability of macrophages, we used Annexin V/7AAD staining to examine the apoptosis of primary peritoneal macrophages after melatonin and/or CpG-ODN treatment. As shown in Fig. 3A,B, 1 mM melatonin did not increase the apoptotic cell percentage of macrophages irrespective of CpG-ODN stimulation, indicating that melatonin had no cytotoxic effects on macrophages and the suppression of TLR9-mediated proinflammatory cytokines production could not be attributable to a direct cytotoxic effect by melatonin.

### Melatonin membrane receptors MT1 and MT2 are dispensable for the suppressive effects of melatonin

**Figure 4:**
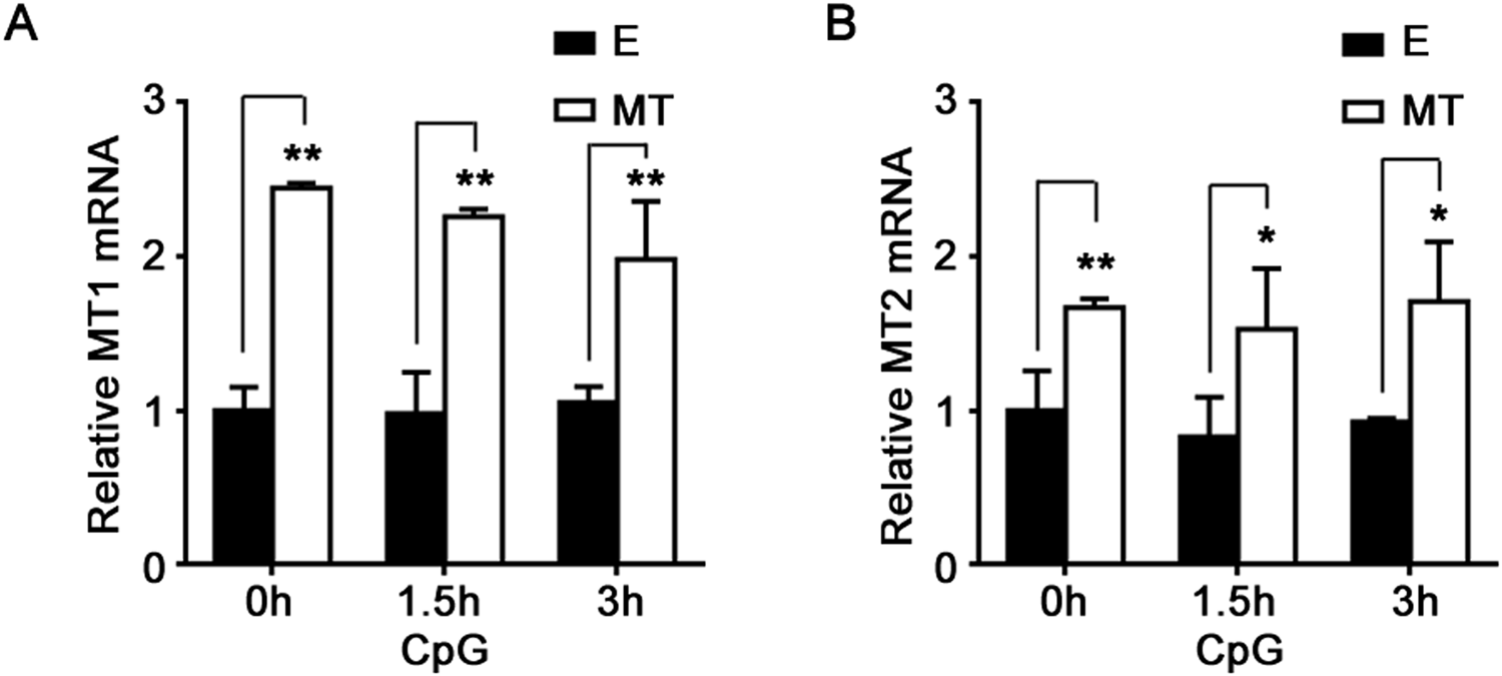
Melatonin upregulates melatonin membrane receptors MT1 and MT2 expressions in peritoneal macrophages. Peritoneal macrophages were pre-treated with 1 mM MT or E for 1 hour and then stimulated with 2 μg/ml CpG-ODN for indicated times. Melatonin membrane receptors MT1 (**A**) and MT2 (**B**) mRNA expressions were detected by real-time PCR. Data are shown as mean ± s.d. (n = 3). *P < 0.05, **P < 0.01 (Student’s t-test). Similar results were obtained in three independent experiments.

**Figure 5:**
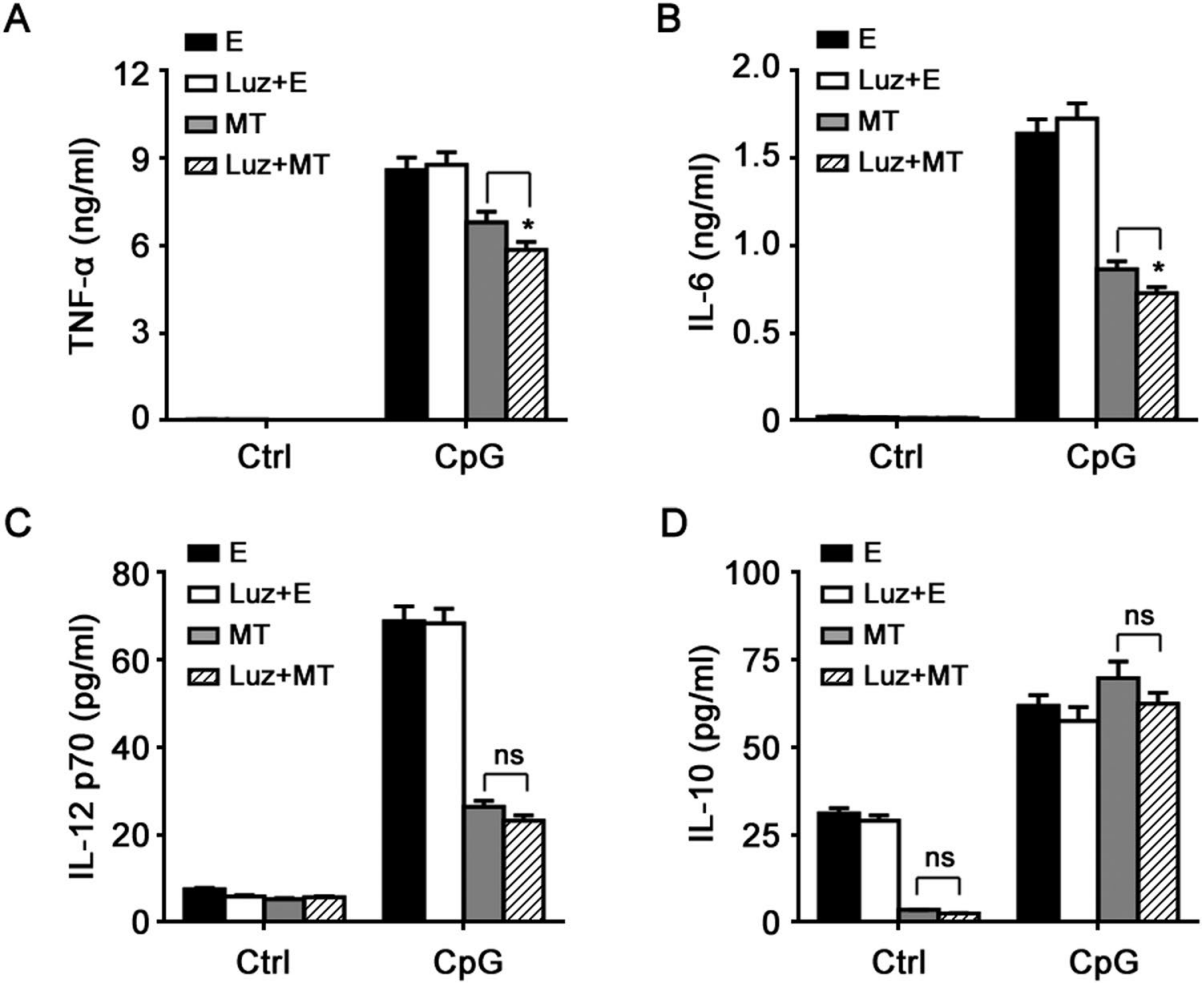
MT1 and MT2 are dispensable for the suppressive effects of melatonin on TLR9 signaling. Peritoneal macrophages were incubated with 10 μM luzindole (a nonselective antagonist of melatonin membrane receptors MT1/MT2) for 1 hour and then treated with 1 mM MT or E for 1 hour. After that, cells were stimulated with 2 μg/ml CpG-ODN for 24 hours. TNF-α (**A**), IL-6 (**B**), IL-12 p70 (**C**) and IL-10 (**D**) in the supernatants were assayed by ELISA. Data are shown as mean ± s.d. (n = 3). “ns” indicates no significance, *P < 0.05 (ANOVA). Similar results were obtained in three independent experiments.

Melatonin exerts a variety of physiological and pharmacological effects on the immune system through activation of two melatonin membrane receptors MT1 and MT2, which belong to G protein-coupled receptors (GPCRs)^19^. We found that peritoneal macrophages expressed melatonin membrane receptors MT1 and MT2, and melatonin treatment upregulated mRNA expressions of MT1 and MT2 in macrophages, whereas CpG-ODN stimulation did not affect mRNA expressions of MT1 and MT2 (Fig. 4A,B). To investigate whether MT1 and MT2 were required for the suppressive effects of melatonin on TLR9-mediated inflammatory cytokines production, luzindole, a nonselective antagonist of both MT1 and MT2, was used. As shown in Fig. 5A–D, routine concentration of luzindole (10 μM)^20,21^ did not reverse the inhibitory effects of melatonin on CpG-ODN-induced TNF-α, IL-6 and IL-12 p70 production, and even further enhanced the suppression function of melatonin on TLR9-mediated TNF-α and IL-6 production. These results demonstrated that melatonin membrane receptors MT1 and MT2 were dispensable for the suppressive effects of melatonin on TLR9-mediated inflammatory cytokines production in macrophages.

### Melatonin inhibits TLR9 ligand-induced ERK1/2 and AKT activation

**Figure 6:**
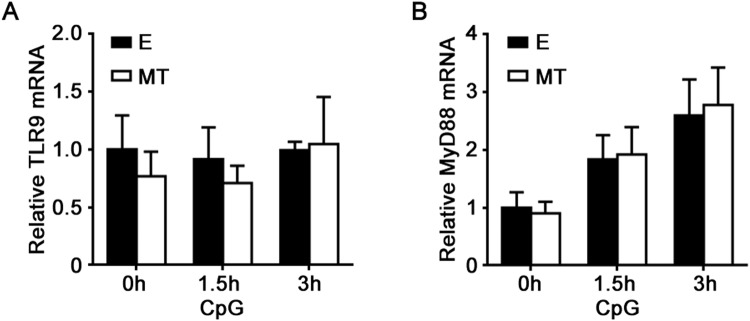
The effects of melatonin on mRNA expressions of TLR9 and MyD88 in peritoneal macrophages. Peritoneal macrophages were pre-treated with 1 mM MT or E for 1 hour and then stimulated with 2 μg/ml CpG-ODN for indicated times. TLR9 (**A**) and MyD88 (**B**) mRNA expressions were detected by real-time PCR. Data are shown as mean ± s.d. (n = 3). Similar results were obtained in three independent experiments.

**Figure 7:**
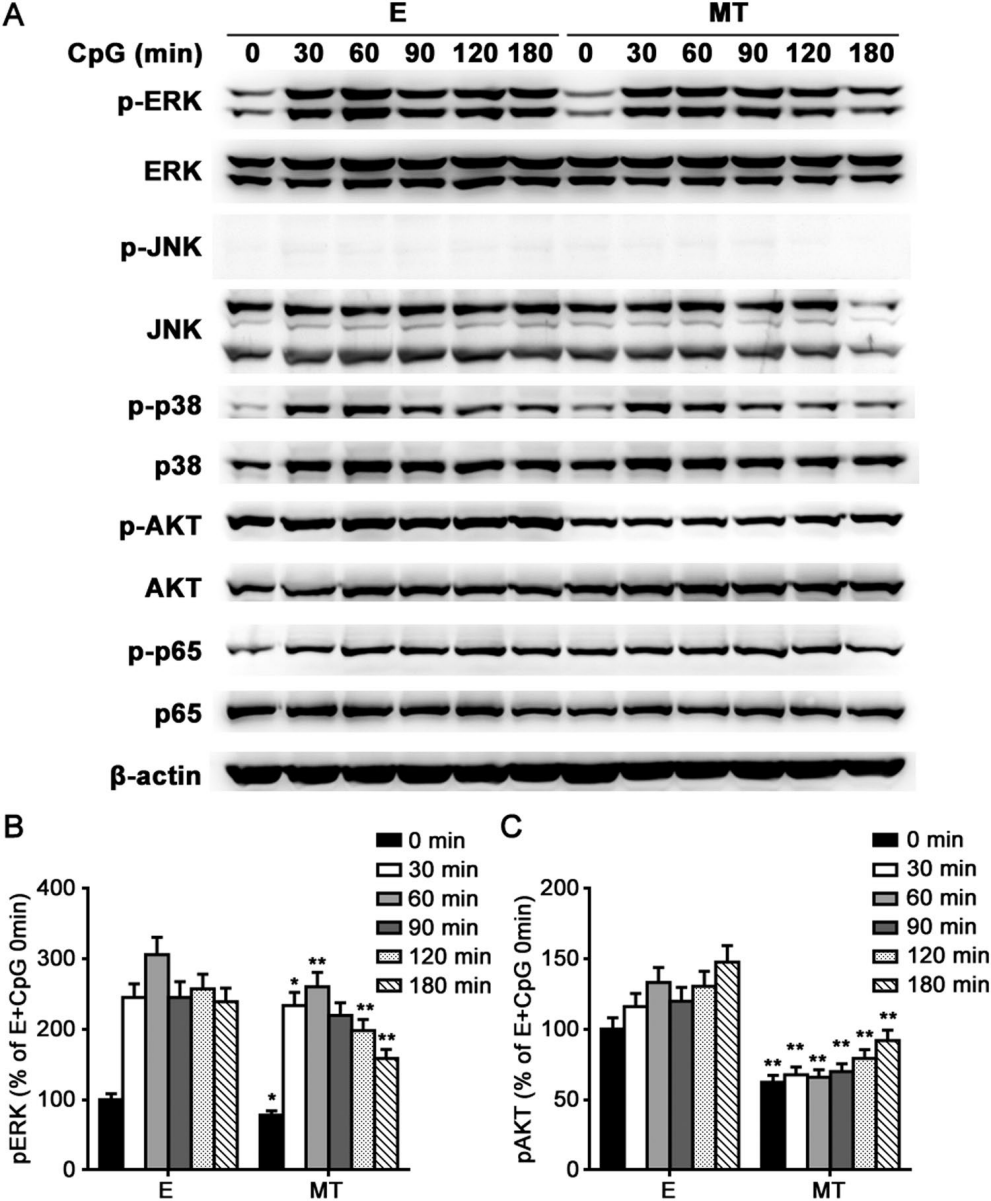
Melatonin inhibits TLR9-mediated ERK1/2 and AKT activation in peritoneal macrophages. Peritoneal macrophages were pre-treated with 1 mM MT or E for 1 hour and then stimulated with 2 μg/ml CpG-ODN for indicated times. Phosphorylated and total ERK1/2, JNK, p38, AKT and NF-κB p65 and β-actin were detected by western blot. Representative images are shown (**A**) and relative expression levels of phosphorylated ERK1/2 and AKT in various groups are shown by the percentage of their expression levels in “E + CpG 0 min” group (**B,C**). *P < 0.05, **P < 0.01 (ANOVA, MT groups in various times are compared to E groups in the same time). Data are representative of three independent experiments with similar results.

TLR9 agonist CpG-ODN induces proinflammatory cytokines production by activating MyD88-dependent MAPK, PI-3K/AKT and NF-κB pathways^1,22^. To investigate the mechanism by which melatonin suppressed CpG-ODN-induced inflammatory cytokines production, we first detected its effects on TLR9 and MyD88 expression, and found that 1 mM melatonin treatment did not affect mRNA expression of TLR9 and MyD88 in primary peritoneal macrophages, irrespective of CpG-ODN stimulation (Fig. 6A,B). We then studied melatonin’s effects on CpG-ODN-induced MAPK, AKT and NF-κB activation in peritoneal macrophages. As shown in Fig. 7A, 2 μg/ml CpG-ODN remarkably induced ERK1/2, p38, AKT, and NF-κB p65 phosphorylation while it did not induce JNK phosphorylation. Interestingly, melatonin selectively attenuated CpG-ODN-induced ERK1/2 and AKT phosphorylation, but had no effects on p38 and NF-κB p65 phosphorylation (Fig. 7A–C). These results suggested that melatonin suppressed CpG-ODN-induced inflammatory cytokines production probably by inhibiting ERK1/2 and AKT activation.

### Melatonin inhibits TLR9-mediated proinflammatory cytokines production *in vivo*

**Figure 8:**
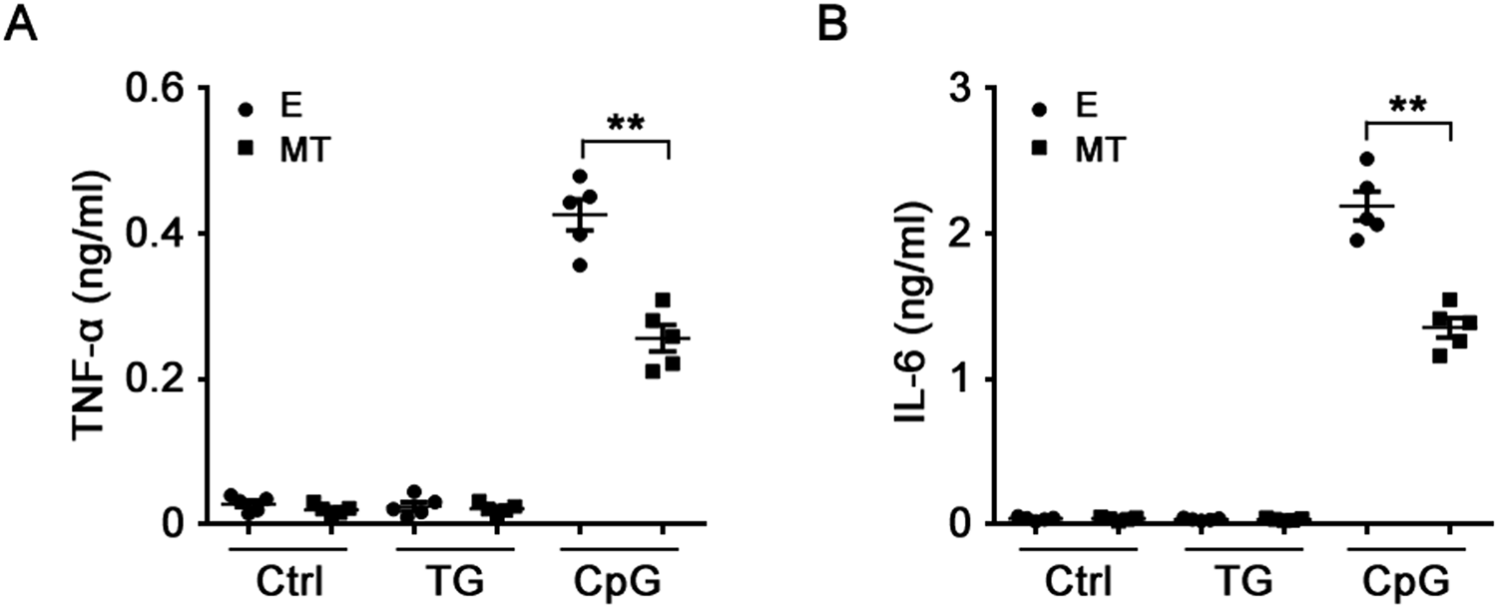
Melatonin inhibits TLR9-mediated proinflammatory cytokines production *in vivo*. C57BL6/J mice were intraperitoneally injected with thioglycollate (TG) to elicite macrophages on day 0 and then received 5 mg/kg melatonin (MT) or ethanol (E) on day 3 and day 4. On day 4, mice were further stimulated by intraperitoneal injection of 50 μg CpG-ODN two hours after melatonin pretreatment. TNF-α (**A**) and IL-6 (**B**) in serum of mice in various groups (n = 5) were assayed by ELISA 2 hours after CpG-ODN injection. Error bars represent s.d. **P < 0.01 (ANOVA).

To explore the role of melatonin in regulating TLR9 signaling in macrophages *in vivo*, C57BL/6J mice were intraperitoneally injected with CpG-ODN in the presence or absence of melatonin pretreatment after mobilizing peritoneal macrophages through thioglycollate (TG) treatment, and then the levels of TNF-α and IL-6 in mouse serum were detected. As shown in Fig. 8A,B, CpG-ODN induced high levels of TNF-α and IL-6 in serum and melatonin pretreatment significantly inhibited CpG-ODN-induced TNF-α and IL-6 production in serum, indicating that melatonin inhibited TLR9-mediated proinflammatory cytokines production *in vivo*.

## Discussion

Increasing evidences show that melatonin not only exerts an immunosuppressive effect on macrophages but also exerts an immunostimulatory effect on macrophages, possibly depending on the manners of melatonin administration or the dose of melatonin^13^. For example, it has been reported that melatonin stimulated a significant increase of IL-12 production in human synovial macrophages and THP-1 cells, however, a significant reduction of IL-12 production was observed when macrophages were previously activated with LPS and then treated with melatonin^23^. Regarding TLRs signaling, it has been demonstrated that melatonin mainly exerts a suppressive effect on TLRs signaling, including TLR3 and TLR4 signalings^8–10,13^. In the present study, we demonstrated for the first time that melatonin suppressed TLR9-mediated inflammatory responses in macrophages, indicating that melatonin was a negative regulator of TLR9-mediated innate immunity in macrophages. Although melatonin upregulated melatonin membrane receptors MT1 and MT2 mRNA expression in macrophages, MT1 and MT2 were dispensable for the suppressive effects of melatonin on TLR9-mediated inflammatory responses. Furthermore, melatonin did not affect TLR9 and MyD88 mRNA expression, but possibly attenuated ERK1/2 and AKT activation to suppress TLR9 signaling.

After stimulation with TLR ligands such as LPS, poly I:C or CpG-ODN, activated macrophages produce various cytokines including TNF-α, IL-6, IL-12, IL-1β, IL-10, and type 1 interferon including IFN-α and IFN-β. TLR9 ligand CpG-ODN induces macrophages and dendritic cells to secret TNF-α, IL-6, IL-12, IL-10 and IFN-α/β (mainly produced by plasmacytoid dendritic cells)^24,25^. The present study demonstrated that melatonin suppressed the mRNA and protein expression of TNF-α, IL-6 and IL-12 p70 induced by CpG-ODN, indicating that melatonin could suppress TLR9-mediated proinflammatory cytokines production in macrophages. Similarly, previous studies showed that melatonin suppressed LPS-induced TNF-α, IL-6, IL-1β, and IL-8 expression in RAW264.7 cells^9,10^. IL-10 induced by CpG-ODN, an anti-inflammatory cytokine, could suppress IL-12 p70 production in macrophages^26^. Our present study demonstrated that melatonin did not suppress or promote IL-10 protein production induced by CpG-ODN, which is different from another study showing that melatonin suppressed LPS-induced IL-10 expression^10^. This indicates that IL-10 is not involved in the inhibitory effects of melatonin on TLR9-mediated inflammatory responses in macrophages.

The molecular mechanisms of action of melatonin mainly include the following four models: (i) via its interaction with membrane receptors such as MT1 and MT2 (at low concentration), (ii) by binding to its nuclear receptors such as RORα and RZR (at low concentration), (iii) via an interaction with cytoplasmic proteins such as calmodulin and hydroquinone (at high concentration), and (iv) via directly, receptor-independent actions such as scavenging ROS/RNS^13,15,27^. It has been shown that the effects of melatonin on immune system are mediated through melatonin receptor-dependent or -independent mechanisms. In multiple sclerosis (MS), melatonin blocks Th17 differentiation and boosts the generation of IL-10-secreting Treg cells by an MT1-dependent mechanism^28,29^. Melatonin inhibits the infectivity of Leishmania by murine peritoneal macrophages and enhances the phagocytosis of zymosan by human colostrum mononuclear cells, both of which are attenuated by luzindole (an antagonist of MT1 and MT2), indicating the involvement of melatonin membrane receptors in these effects^30,31^. The present study showed that luzindole did not reverse the inhibitory effects of melatonin on TLR9-mediated inflammatory responses in macrophages, so melatonin exerts its inhibitory effects on TLR9 signaling through melatonin membrane receptor-independent way.

Given that melatonin at high concentration also exerts its effects through receptor-independent manners in addition to its interaction with either melatonin membrane or nuclear receptors^13,15,27^, in the present study, high-concentration melatonin (reaching the level of millimole) probably directly regulated the expression or activation of TLR9 signal molecules, similar to its mechanisms in regulating TLR3 or TLR4 signaling in macrophages^8–10^. Melatonin decreased TLR-3-mediated TNF-α and iNOS mRNA expression in respiratory syncytial virus-infected RAW264.7 cells via inhibition of NF-κB activation without influencing TLR3 and MyD88 mRNA expression^8^. In LPS-stimulated RAW264.7 cells, melatonin attenuated the expression of MyD88 and the activation of NF-κB, and also inhibited TLR4-mediated AKT phosphorylation and attenuated the elevation of IRF3, which was involved in TLR4-mediated TRIF-dependent signaling pathway^10^. In another study, melatonin inhibited LPS induced production of nitric oxide and IL-6 in RAW264.7 cells by suppressing NF-κB and STAT1 activation^9^. In the present study, melatonin inhibited CpG-ODN-induced ERK1/2 and AKT activation without affecting NF-κB and p38 activation in macrophages. Furthermore, melatonin did not affect TLR9 and MyD88 mRNA expression. Hence, melatonin might suppress TLR9-mediated innate immune responses through inhibiting ERK1/2 and AKT activation. This study, together with other previous studies, indicate that melatonin regulates TLR signaling in macrophages through different mechanisms, dependent on different TLR subtypes and their ligands^8–10^. It remains to be further investigated how melatonin regulates TLR9-mediated ERK1/2 and AKT activation in macrophages. In our previous study, MEKK2 knockdown (specifically inhibiting ERK1/2 activation) significantly suppressed CpG-ODN-induced TNF-α and IL-6 production in macrophages^4^. In addition, other studies also showed that wortmannin, a PI-3K inhibitor, inhibited the production of IL-12 induced by CpG DNA in bone marrow derived dendritic cells^32^, and another PI-3K inhibitor, LY294002, also reduced TNF-α production induced by CpG DNA in macrophages^33^. These studies further support that ERK1/2 and AKT activation is important for TLR9-mediated inflammatory cytokines production in macrophages.

There is only one published research reporting on the relationship between melatonin and TLR9 signaling. This study showed that JNK-enhanced TLR9 signal pathway mediated allergic airway inflammation through suppressing melatonin biosynthesis^34^. However, there is no research addressing the regulatory function of melatonin on TLR9 signaling. Our present study showed for the first time that melatonin regulated TLR9-mediated innate immune responses and TLR9 signaling pathways.

TLR9-mediated innate immunity and inflammation plays important roles in infectious diseases, autoimmune diseases and cancer^25,35^. Previous studies showed that polymicrobial sepsis in mouse experimental peritonitis model induced by caecal ligation and puncture (CLP) was TLR9-dependent^36,37^. It has been demonstrated that melatonin protects mice against septic shock or septic organs injury in CLP sepsis models through different mechanisms: these include antioxidative effects, anti-nitrosative effects, protective effects against mitochondrial dysfunction and anti-inflammatory effects^18,38–43^. Hence, it is possible that melatonin also provides protections against sepsis through suppressing TLR9-mediated inflammation, which requires to be further investigated. Furthermore, our present study demonstrated that *in vivo* administration of melatonin exerted notable suppressive effects on TLR9–mediated inflammatory cytokines production in mice, further raising this possibility.

Taken together, our study demonstrated that melatonin suppressed TLR9-triggered proinflammatory TNF-α, IL-6 and IL-12 p70 production in macrophages not through melatonin membrane receptors-dependent manners but probably through inhibiting TLR9-mediated ERK1/2 and AKT activation. These results proved that melatonin was a negative regulator of TLR9-mediated innate immunity in macrophages.

## Materials and Methods

### Mice and reagents

C57BL/6J mice (female, 6–8 weeks old) were obtained from Joint Ventures Sipper BK Experimental Animal Company (Shanghai, China). All animal experiments were performed in accordance with the National Institutes of Health Guide for the Care and Use of Laboratory Animals, with the approval of the Scientific Investigation Board of Second Military Medical University (Shanghai, China). Melatonin (M5250), 7-aminoactinomycin (7AAD, A9400) and luzindole (L2407) were from Sigma-Aldrich (St Louis, MO). Phosphorothioate-modified CpG-ODN 1668 (5′-TCC ATG ACG TTC CTG ATG CT-3′) was synthesized by Sangon Biotech Company (Shanghai, China). Endotoxin in these ODNs was removed using Endotoxin Removal Solution (Sigma-Aldrich) as described previously^44^, and the endotoxin level was <0.015 U/mg CpG-ODN. Antibodies specific for ERK1/2 (9107), phosphorylated ERK1/2 (9101), JNK (9252), phosphorylated JNK (9255), p38 (9212), phosphorylated p38 (9211), AKT (9272), phosphorylated AKT (4060), p65 (3034), and phosphorylated p65 (3033) were from Cell Signaling Technology. Annexin V apoptosis detection kit APC was from eBioscience (ThermoFisher scientific). Antibody specific for β-actin (sc-1616) and HRP-coupled secondary antibody were from Santa Cruz.

### Cell culture and treatments

Thioglycollate-elicited mouse peritoneal macrophages were isolated and cultured as previously described^4^. C57BL/6J mice (6–8 weeks old) were intraperitoneally injected with 1 ml of 3% sodium thioglycollate (Merck) solution per mouse. After 3 days, cells in the abdominal cavity were collected by lavaging with 10–15 ml FBS-free DMEM and centrifugated. Cells were cultured and maintained in DMEM with 10% (vol/vol) FBS for 24 hours. After removing the floating cells, adhered peritoneal macrophages were subjected for further experiments. The cells were pre-treated with various concentrations of melatonin (stock concentration 200 mM, dissolved in ethanol) for 1 hour and then stimulated with 2 μg/ml CpG-ODN for indicated times (Supplementary Fig. 1). In some experiments, the cells were incubated with 10 μM luzindole for 1 hour before pretreatment with melatonin (Supplementary Fig. 1C).

### Detection of cytokine production

The concentrations of TNF-α, IL-6, IL-12p70 and IL-10 in culture supernatants or serum were measured with ELISA kits from R&D systems (Minneapolis, MN) according to the manufacturer’s instructions.

### Detection of cell apoptosis

After treatment with melatonin and/or CpG-ODN (Supplementary Fig. 1B), peritoneal macrophages were harvested and stained with Annexin V-APC and 7AAD according to the manufacturer’s instructions. Then the apoptosis of macrophages was assayed by flow cytometry (LSR II, BD biosciences) and the data were analyzed with FlowJo Version 5.7.2 software (Tree Star).

### Quantitative reverse transcription-PCR

Total RNA was extracted with RNA extraction kit (RNAfast200, Fastagen Biotech Co., China) and reversed-transcripted with reverse transcriptase M-MLX (Takara) following the manufacturer’s instructions. Real-time PCR was performed with a SYBR RT-PCR kit (Takara) on LightCycler system (Roche). Data were normalized by the level of β–actin expression in each individual sample. 2^−△△Ct^ method was used to calculate relative expression changes. The sequences of the primers used were from PrimerBank as follows: β–actin sense, 5′-GGCTGTATTCCCCTCCATCG-3′ and antisense, 5′-CCAGTTGGTAACAATGCCATGT-3′; MT1 sense, 5′-TACACTGTCAAGTCAGCGCAT-3′ and antisense, 5′AGCAGTAACGGTTCATAGCGA-3′; MT2 sense, 5′-GAACAGCTCAATCCCTAACTGC-3′ and antisense, 5′-ACGACTACTGTAGATAGCATGGG-3′; TNF-α sense, 5′-CCCTCACACTCAGATCATCTTCT-3′ and antisense, 5′-GCTACGACGTGGGCTACAG-3′; IL-6 sense, 5′-TAGTCCTTCCTACCCCAATTTCC-3′ and antisense, 5′-TTGGTCCTTAGCCACTCCTTC-3′; IL-12 p35 sense, 5′-CTGTGCCTTGGTAGCATCTATG-3′ and antisense, 5′-GCAGAGTCTCGCCATTATGATTC-3′; IL-12 p40 sense, 5′-TGGTTTGCCATCGTTTTGCTG-3′ and antisense, 5′-ACAGGTGAGGTTCACTGTTTCT-3′; IL-10 sense, 5′-GCTCTTACTGACTGGCATGAG-3′ and antisense, 5′-CGCAGCTCTAGGAGCATGTG-3′; IFN-β sense, 5′-CAGCTCCAAGAAAGGACGAAC-3′ and antisense, 5′-GGCAGTGTAACTCTTCTGCAT-3′; TLR9 sense, 5′-ATGGTTCTCCGTCGAAGGACT-3′ and antisense, 5′-GAGGCTTCAGCTCACAGGG-3′; MyD88 sense, 5′-TCATGTTCTCCATACCCTTGGT-3′ and antisense, 5′-AAACTGCGAGTGGGGTCAG-3′.

### Western blot

After stimulated with 2 μg/ml CpG-ODN for indicated times (Supplementary Fig. 1D), primary peritoneal macrophages were washed twice with ice-cold PBS and lysed with M-PER Protein Extraction Reagent (Pierce) supplemented with protease inhibitor cocktail, and then protein concentrations of the extracts were measured with BCA assay according to the manufacturer’s instructions (Pierce). Equal amounts of extracts were loaded for SDS–PAGE, transferred onto nitrocellulose membranes and then blotted with indicated antibodies, as described previously^4^. Finally, signal intensity was determined using the Tanon 5200S Chemiluminescent Imaging System (Tanon). Images had been cropped for presentation. Full-size images were presented in Supplementary Fig. 2.

### Induction of proinflammatory cytokines by CpG-ODN *in vivo*

C57BL/6J mice (6–8 weeks old) were intraperitoneally injected with 1 ml of 3% sodium thioglycollate (Merck) solution on day 0. On day 3 and day 4, mice were intraperitoneal injected with 5 mg/kg melatonin. On day 4, mice were stimulated by intraperitoneal injection of 50 μg CpG-ODN 2 hours after melatonin injection (Supplementary Fig. 1E). Two hours after CpG-ODN injection, the serum samples were collected and used for ELSIA assays of TNF-α and IL-6.

### Statistical analysis

All data were presented as means ± standard deviation (S.D.). The statistical significance was calculated by one-way ANOVA test or two-tailed Student’s t test using Prism software 5 (GraphPad Software, Inc., San Diego, CA). *P* value less than 0.05 was considered statistically significant.

## Electronic supplementary material

Supplementary Figure 1 and 2

## Data Availability

All data generated or analyzed during this study are included in this manuscript.
