# Supplementary material for: Melatonin suppresses TLR9-triggered proinflammatory cytokine production in macrophages by inhibiting ERK1/2 and AKT activation
Source: Sci Rep. 2018 Oct 22;8:15579. doi: 10.1038/s41598-018-34011-8 (PMC6197220; doi:10.1038/s41598-018-34011-8)
Supplement: Supplementary file 1 — Supplementary Figure 1 and 2 [file 41598_2018_34011_MOESM1_ESM.pdf]

Melatonin suppresses TLR9-triggered proinflammatory cytokine production in macrophages by inhibiting ERK1/2 and AKT activation

Xiongfei Xu[1,2,*] | Guoquan Wang[3,*] | Lingling Ai[4,*] | Jianhui Shi[1] | Jing Zhang[5] | Yu-Xia Chen[1]

[1]Department of Pathophysiology, Second Military Medical University, Shanghai, 200433, China.

[2]Department of Hepatobiliary Pancreatic Surgery, Changhai Hospital, Second Military Medical University, Shanghai, 200433, China.

[3] Department of Neurology, Junkang Hospital, Shanghai, 200125, China.

[4]Department of Otolaryngology, No. 455 Hospital of PLA, Shanghai, 200052, China.

[5]Department of Pathology, Changhai Hospital, Second Military Medical University, Shanghai, 200433, China.

*These authors equally contributed to this work.

Correspondence

Jing Zhang, Email: zhangjing@smmu.edu.cn. Or Xiongfei Xu, Email: xiongfeixu@126.com.

Supplementary Fig. 1

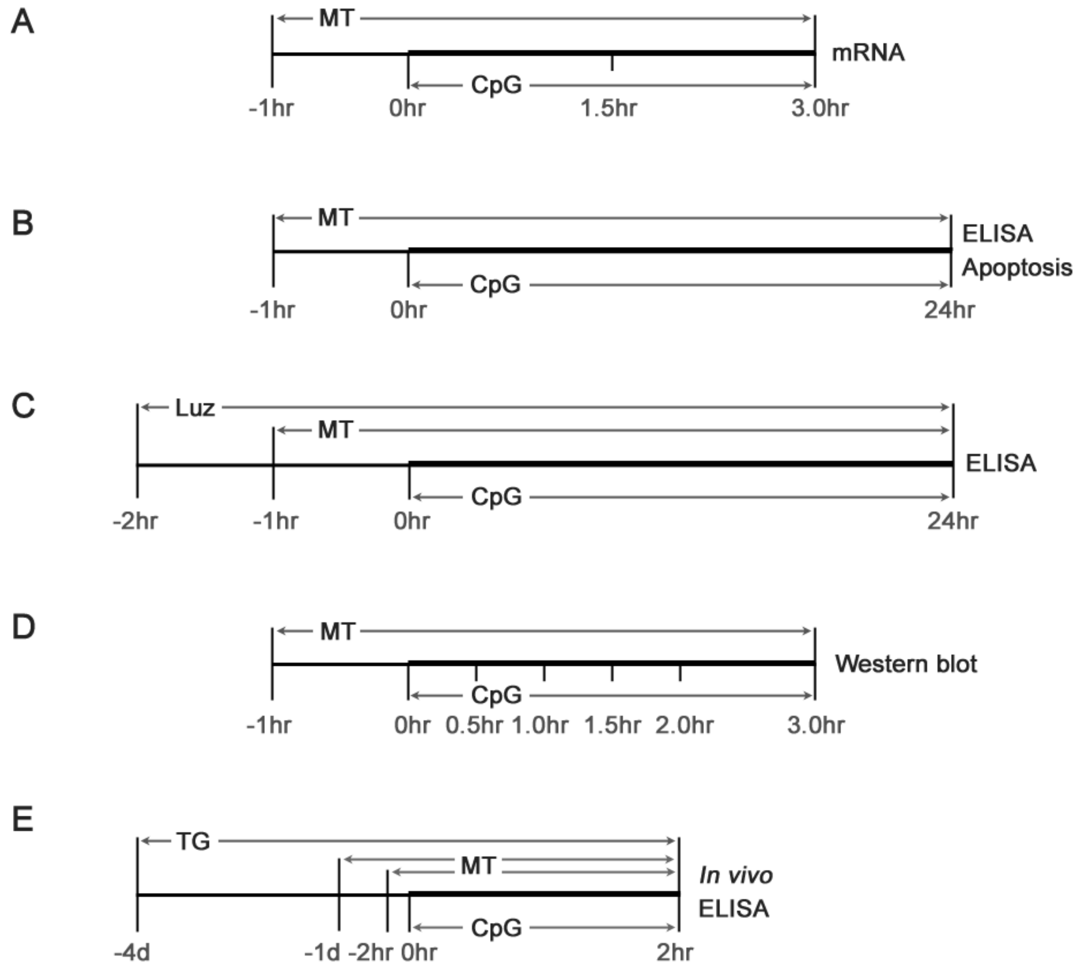

Supplementary Figure 1. **The study design for investigating the effects of melatonin on TLR9 ligand-induced inflammatory responses in macrophages.**

(A) To investigate the effects of melatonin (MT) on mRNA expressions of inflammatory cytokines induced by TLR9 ligand, peritoneal macrophages were pre-treated with MT and then stimulated with CpG-ODN for 1.5 or 3.0 hours. Real-time PCR was used to assay mRNA expression. (B) To investigate the effects of MT on inflammatory cytokines production induced by TLR9 ligand and cell viability, peritoneal macrophages were pre-treated with MT and then stimulated with CpG-ODN for 24 hours. ELISA and flow cytometry were used to assay cytokines production and cell apoptosis, respectively. (C) To investigate whether melatonin membrane receptors were involved in the effects of MT on

inflammatory cytokines production induced by TLR9 ligand, a melatonin membrane receptor antagonist, luzindole was used. (D) To investigate the effects of MT on signaling pathways induced by TLR9 ligand, peritoneal macrophages were pre-treated with MT and then stimulated with CpG-ODN for various times. Western blot was used to assay signaling proteins activation. (E) To investigate the effects of MT on TLR9-mediated proinflammatory cytokines production in macrophages in vivo, C57BL/6J mice were intraperitoneally injected with thioglycollate (TG) to elicite macrophages and then pretreated with 5 mg/kg MT. Proinflammatory cytokines in serum were assayed by ELISA two hours after intraperitoneal injection of 50μg CpG-ODN.

Supplementary Fig. 2

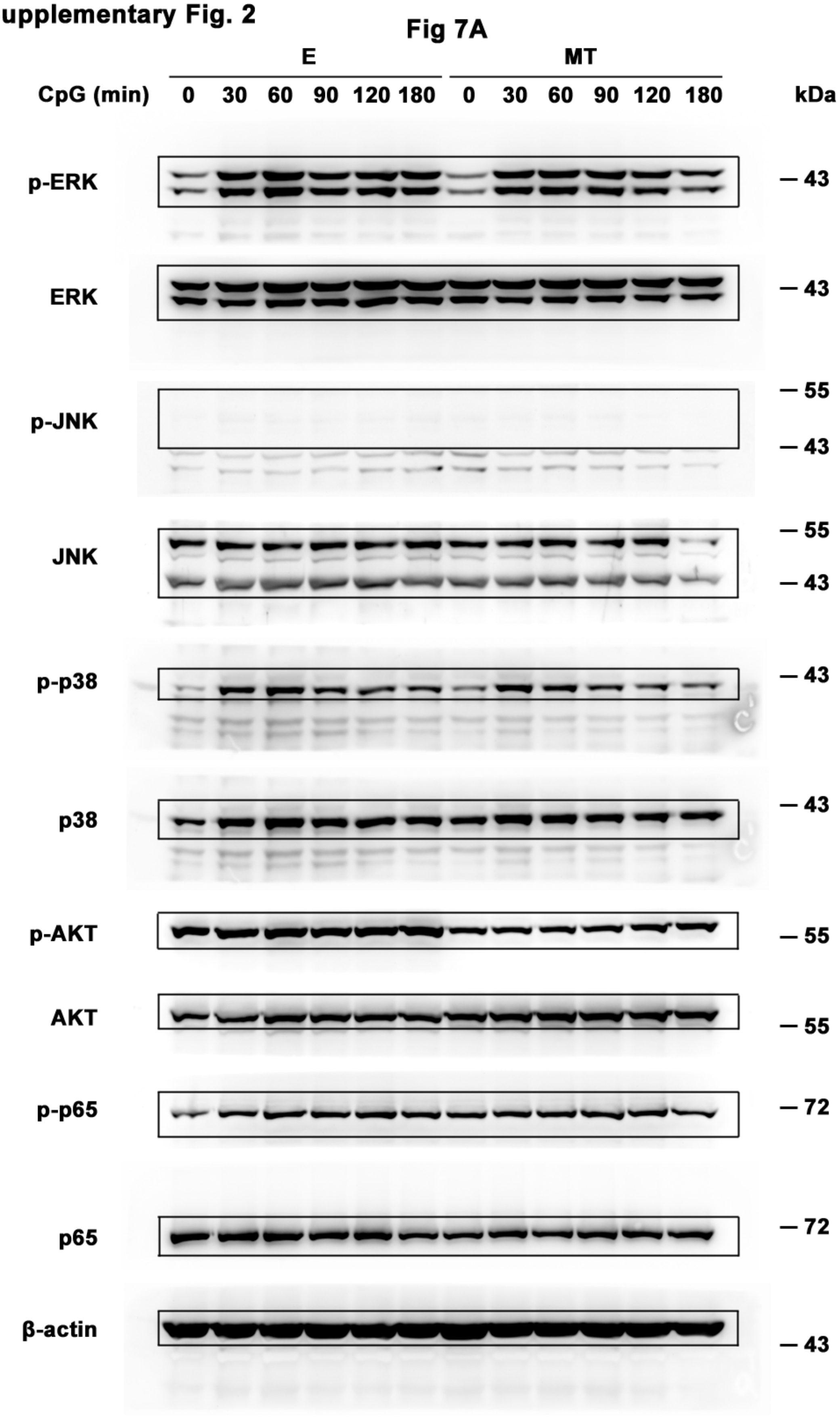

Supplementary Figure 2. Full-size images of cropped images in Figure 7A.
